# Comparison of Transanal Endoscopic Microsurgery and Total Mesorectal Excision in the Treatment of T1 Rectal Cancer: A Meta-Analysis

**DOI:** 10.1371/journal.pone.0141427

**Published:** 2015-10-27

**Authors:** Jun-Yang Lu, Guo-Le Lin, Hui-Zhong Qiu, Yi Xiao, Bin Wu, Jiao-Lin Zhou

**Affiliations:** Department of General Surgery, Peking Union Medical College Hospital, Chinese Academy of Medical Sciences and Peking Union Medical College, Beijing, China 100730; The University of Texas MD Anderson Cancer Center, UNITED STATES

## Abstract

**Background:**

Transanal endoscopic microsurgery (TEM) for the treatment of early-stage rectal cancer has attracted attention due to its advantages of reduced surgical trauma, fewer complications, low operative mortality, rapid postoperative recovery and short hospital stay. However, there are still significant controversies regarding TEM for the treatment of rectal cancer, mainly related to the prognosis associated with this method.

**Objective:**

This study sought to compare the efficacy of transanal endoscopic microsurgery (TEM) and total mesorectal excision (TME) for the treatment of T1 rectal cancer.

**Methods:**

We searched the Cochrane Library, PubMed, Embase and CNKI databases. Based on the Cochrane Handbook for Systematic Reviews, we screened the trials, evaluated the quality and extracted the data.

**Results:**

One randomized controlled trial (RCT) and six non-randomized controlled clinical trials (CCTs) were included in the meta-analysis (a total of 860 rectal cancer patients were included; 303 patients were treated with TEM, and 557 patients were treated with TME). Analysis revealed that all seven studies reported local recurrence rates, and there was a significant difference between the TEM and TME groups [odds ratio (OR) = 4.62, 95% confidence interval (CI) (2.03, 10.53), P = 0.0003]. A total of five studies reported distant metastasis rates, and there was no significant difference between the TEM and TME groups [OR = 0.74, 95%CI (0.32, 1.72), P = 0.49]. A total of six studies reported postoperative overall survival of the patients, and there was no significant difference between the TEM and TME groups [OR = 0.87, 95%CI(0.55, 1.38), P = 0.55]. In addition, two studies reported the postoperative disease-free survival rates of patients, and there was no significant difference between the TEM and TME groups [OR = 1.12, 95%CI (0.31, 4.12), P = 0.86].

**Conclusions:**

For patients with T1 rectal cancer, the distant metastasis, overall survival and disease-free survival rates did not differ between the TEM and TME groups, although the local recurrence rate after TEM was higher than that after TME.

## Introduction

Total mesorectal excision (TME) for rectal cancer has been widely used as the standard surgical treatment for rectal cancer. Combined with preoperative neoadjuvant therapy and postoperative chemotherapy, the long-term survival rates of rectal cancer patients after surgery have been significantly improved [1, 2]. Surgical treatment of rectal cancer aims to improve the patient's quality of life while maintaining the radical nature of the treatment. To a certain extent, TME for rectal cancer can reduce the recurrence rate, but due to the significant trauma associated with this procedure, the rates of postoperative complications and mortality remain high [3, 4]. Transanal endoscopic microsurgery (TEM) for the treatment of early-stage rectal cancer has attracted attention due to its advantages of reduced surgical trauma, fewer complications, low operative mortality, rapid postoperative recovery and short hospital stay [5, 6]. However, there are still significant controversies regarding TEM for the treatment of rectal cancer, mainly related to the prognosis associated with this method. This study sought to conduct a meta-analysis of the efficacies of TEM and TME in the treatment of T1 rectal cancer.

## Materials and Methods

### Literature search

We searched for potentially relevant publications through October 2014. We collected all randomized controlled trials (RCTs) and non-randomized controlled clinical trials (CCTs) that evaluated TEM and TME for the treatment of early rectal cancer. Two authors independently searched the literature, particularly the Cochrane Library, PubMed, Embase and CNKI (Chinese) databases. We queried the databases using both free-text terms and medical subject headings. The English terms used in the query included “rectal cancer”, “rectal neoplasms”, “rectal carcinoma”, “rectal tumor”, “local excision”, “TEM”, “transanal endoscopic microsurgery”, “TME” and “total mesorectal excision”. In addition, the Chinese terms used in the query included “Zhichang Ai” (rectal cancer), “Jubu Qiechu” (local excision), “Jinggang Jubu Qiechu” (TEM), and “Quan Ximo Qiechu” (TME). We also searched all major European and American conference publications, and we checked the references of retrieved articles as a supplement and contacted the authors as well as relevant domestic experts to obtain full-text papers and more complete data.

### Inclusion criteria and exclusion criteria

The inclusion criteria included (1) Published RCTs and CCTs in Chinese or English; (2) Patients were confirmed by pathological examination to have rectal cancer; (3) TNM staging was used in clinical practice, and the stages were T1N0M0; (4) Information such as gender, age, race, tumor size, and long-term survival results was included; (5) The experimental group received TEM, and the control group received TME, including laparoscopic rectal cancer resection and laparotomic rectal cancer resection; (6) The total number of included cases was ≥ 20.

The exclusion criteria were (1) Patients with recurrent rectal cancer; (2) Basic research such as animal experiments.

### Data collection

Two investigators (JYL BW) independently screened the literature according to the inclusion and exclusion criteria. The pre-designed data extraction form was used to obtain the information, and the two investigators cross-checked each item one by one. Disagreement was resolved by discussion. If the research report only contained incomplete information, we then contacted the authors to obtain the rest of the information. If we could not obtain the relevant data, the research report was excluded. The following characters were extracted from each study: author, publication date, number of patients, age, tumor size, location, type of operation, local recurrence rate, distant recurrence rate, overall survival rate and disease-free survival rate.

### Literature quality assessment

Each study was independently evaluated by two investigators using the Newcastle-Ottawa Scale (NOS) [7]. We performed the evaluations according to the three aspects of patient Selection, Comparability of TEM and TME groups, and assessment of Outcome (Table 1). A study can be awarded a maximum of four stars for Selection, two stars for Comparability and three stars for Outcome categories. The quality of each study was graded as either level 1 (0 to 4) or level 2 (5 to 9).

**Table 1:** Checklist for quality assessment and scoring for cohort studies.

Checklist
**Selection**
1 Representativeness of the exposed cohort
a) Truly representative of the community *; b) somewhat representative of the community *; c) selected group of users e.g. nurses, volunteers; d) no description of the derivation of the cohort.
2 Selection of the non exposed cohort
a) Drawn from the same community as the exposed cohort *; b) drawn from a different source; c) no description of the derivation of the non exposed cohort.
3 Ascertainment of exposure
a) Secure record *; b) structured interview *; c) written self report; d) no description.
4 Demonstration that outcome of interest was not present at start of study
a) Yes *; b) no.
**Comparability**
1 Comparability of cohorts on the basis of the design or analysis
a) Study controls for TEM/TME *; b) study controls for any additional factor *.
**Outcome**
1 Assessment of outcome
a) Independent blind assessment *; b) record linkage *; c) self report; d) no description.
2 Was follow-up long enough for outcomes to occur
a) Yes (select an adequate follow up period for outcome of interest) *; b) no.
3 Adequacy of follow up of cohorts
a) Complete follow up *; b) >80% follow up, or description provided of those lost) *; c) follow up rate < 80% and no description of those lost; d) no statement.

* Each option marked with ‘*’ can get one star in the score.

### Statistical analysis

Meta-analysis was performed using the RevMan 5.2 software provided by the Cochrane Collaboration network. Count data utilized the relative risk (RR) or odds ratio (OR) as the effect size, and each effect size was given with the 95% confidence interval (CI) [8]. First, we used the χ2 test to investigate the heterogeneity of the included studies. If there was no heterogeneity or a low level of heterogeneity (I^2^≤50%, P≥0.1), we used a fixed effects model for the meta-analysis, and if I^2^≤50% and P<0.1, we used a random effects model for the meta-analysis. In cases of heterogeneity (I^2^>50%, P<0.1), the cause of the heterogeneity was first analyzed. In the absence of clinical heterogeneity, a random effects model was used for the meta-analysis. In the presence of clinical heterogeneity, only a descriptive analysis was performed. When necessary, a sensitivity analysis was used to test the stability of the results.

## Results

### Literature search results

**Fig 1:**
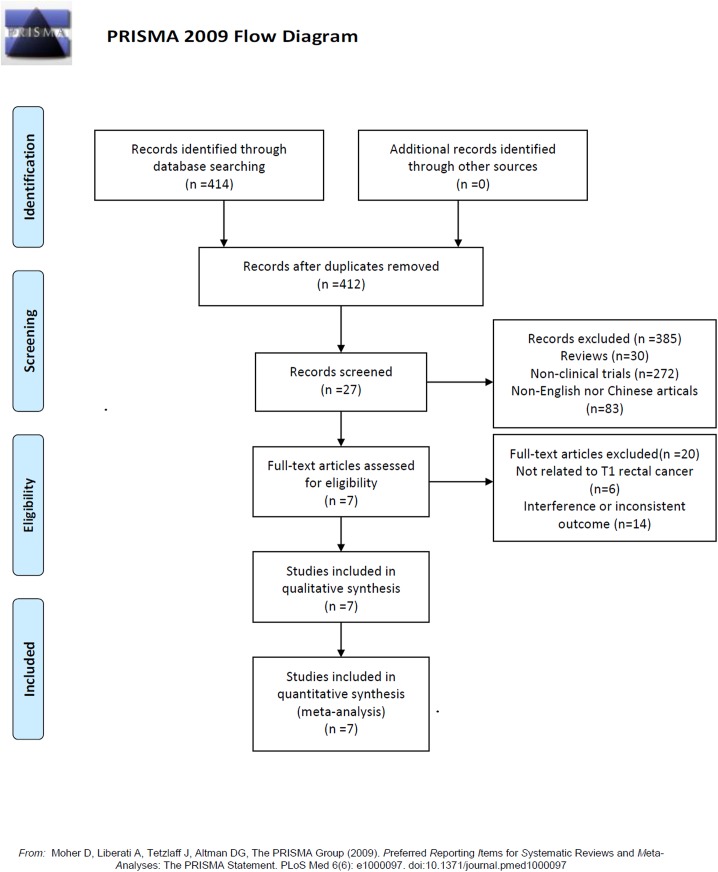
Literature screening flow chart and results.

The initial screen identified 414 articles. After excluding duplicate studies and reading the titles and abstracts of the articles, 385 non-clinical studies and animal experiments were excluded, and 29 studies were preliminarily included. Subsequently, we read the full text and excluded 22 studies that did not meet the inclusion criteria. The final seven studies [9–15] were all English-language studies. The literature screening process and results are shown in Fig 1.

#### Basic characteristics of the included studies

As shown in Table 2, the seven included studies assessed a total of 860 cases of rectal cancer, of which 303 cases received TEM and 557 received TME. Ages ranged from 57.7 years [12] to 71 years [10]. The tumor diameter ranged from 2.35cm [12] to 3.78cm [12], but two studies [11, 15] didn’t describe the tumor size. Distance from anus to the lower edge of tumor ranged from 6.7cm [12] to 10.9cm [9]. Three studies didn’t address the location of the tumor [11, 14, 15]. Duration of follow up varied from 21.6 months [13] to 93 months [9]. The detailed characteristics of the studies are shown in S1 Table.

**Table 2:** Characteristics of the included studies.

Included studies	Country	Study design	Treatment plan	No. of cases
Palma2009[9]	Germany	Cohort study	TEM/TME	34/17
De Graaf2009[10]	Netherlands	Cohort study	TEM/TME	80/75
Ptok2007[11]	U.S.A	Cohort study	TEM/TME	35/359
Lee2003[12]	Korea	Cohort study	TEM/TME	52/17
Langer2003[13]	Germany	Cohort study	TEM/TME	20/18
Heintz1998[14]	Germany	Cohort study	TEM/TME	58/45
Winde1996[15]	Germany	RCT	TEM/TME	24/26

RCT: Randomized-controlled trial; TEM: Transanal endoscopic microsurgery; TME: Total mesorectal excision

#### The quality assessment of the included studies

The quality assessment of the included studies was performed using NOS. Only one trial [11] was multi-center study. In six studies [9, 11–15], the patients of TEM and TME groups were from the same centers. But in the remaining one study [10] the patients of TEM group were from one center and TME group from multi-centers. Six studies [9, 10, 12–15] described the diameters of tumors and there was no significant difference between the TEM and TME groups in five studies [9, 10, 13–15]. Mean time of follow-up was less than 36 months in two studies [12, 13]. The patients lost to follow up were less than 10% in three studies [9, 11, 15]. The remaining four studies did not address the issue of follow up rate [10, 12, 13]. The results of scoring revealed that all the seven studies belonged to level 2, including three of eight-star [9, 11, 15], one of seven-star [14], two of six-star [10, 13] and one of five-star [12]. The detailed results of the quality assessment of the studies are shown in Table 3.

**Table 3:** The quality assessment of the included studies.

Included studies	Selection				Comparability		Outcome			Score
	1	2	3	4	1	2	1	2	3	
Palma2009[9]		*	*	*	*	*	*	*	*	********
De Graaf2009[10]		*	*	*		*	*	*		******
Ptok2007[11]	*	*	*	*	*		*	*	*	********
Lee2003[12]		*	*	*	*		*			*****
Langer2003[13]		*	*	*	*	*	*			******
Heintz1998[14]		*	*	*	*	*	*	*		*******
Winde1996[15]		*	*	*	*	*	*	*	*	********

### Primary outcome results

#### Local recurrence rate

**Fig 2:**
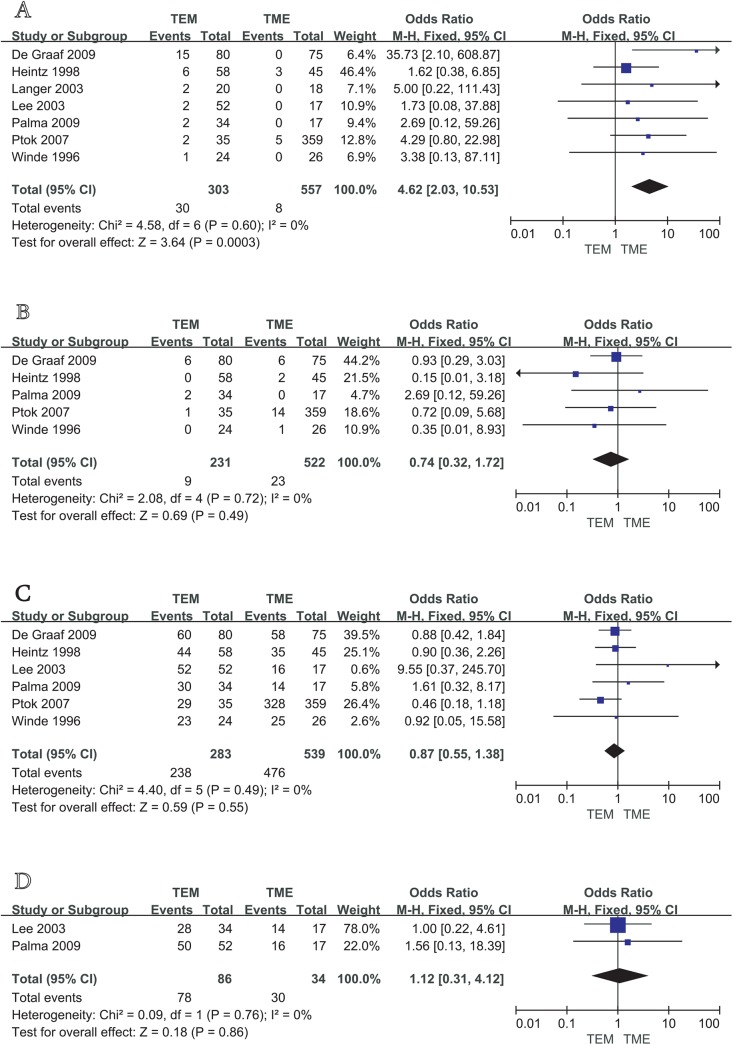
Meta analysis of local recurrence rate (A), distant metastasis rate (B), overall survival (C), disease-free survival (D) between TEM and TME in the treatment of T1 rectal cancer.

The seven studies all reported postoperative local recurrence rates, with fair homogeneity among the studies (P = 0.75, I^2^ = 0%). We therefore used a fixed effects model to perform the meta-analysis. The results showed a statistically significant difference between the TEM and TME groups [OR = 4.62, 95%CI (2.03, 10.53), P = 0.0003], suggesting that for the patients of T1 stage rectal cancer, the rate of local recurrence after TME is significantly lower than that after TEM (Fig 2A).

#### Distant metastasis rate

Five studies [9–11, 14, 15] reported postoperative distant metastasis rates, with no significant heterogeneity among studies (P = 0.72, I2 = 0%). Therefore, a fixed effects model was used to perform the meta-analysis. The results showed that the differences between the two groups were not statistically significant [OR = 0.74, 95%CI (0.32, 1.72), P = 0.49], suggesting that there was no significant difference in the distant metastasis rate between TEM and TME in the treatment of T1 rectal cancer (Fig 2B).

#### Overall survival

Six studies [9–12, 14, 15] reported that postoperative overall survival rates of patients, with no significant heterogeneity among studies (P = 0.49, I2 = 0%). Therefore, a fixed effects model was used to conduct the meta-analysis. The results showed that the differences between the two groups were not statistically significant [OR = 0.87, 95%CI (0.55, 1.38), P = 0.55], suggesting that there was no significant difference in overall survival between TEM and TME in the treatment of T1 rectal cancer (Fig 2C).

#### Disease-free survival

Two studies [9, 12] reported the disease-free survival rates of patients, with no significant heterogeneity between studies (P = 0.76, I2 = 0%). Thus, a fixed effects model was used to conduct the meta-analysis. The results showed that the difference between the TEM and TME groups was not statistically significant [OR = 1.12, 95%CI (0.31, 4.12), P = 0.86], suggesting that TEM and TME did not differ significantly in terms of their impact on disease-free survival in T1 stage rectal cancer patients (Fig 2D).

#### Meta-regression

Meta-regression was conducted to explore the possible causes of heterogeneity. Meta-regression analysis demonstrated that the included studies had fair homogeneity. Age of patients (P = 0.333), number of included cases (P = 0.941), follow-up time (P = 0.786) and study type (P = 0.579) had no effect on heterogeneity. The detailed results of meta-regression are shown in S2 Table.

#### Publication bias

We selected the rates of local recurrence, distant metastasis and overall survival to conduct the publication bias analysis. Because there were fewer included studies on disease-free survival, publication bias analysis was not performed. The results showed that the funnel plot was generally symmetric, and the publication bias was therefore small.

## Discussion

Radical resection (TME) is considered the best method for the treatment of rectal cancer, as studies have shown that the postoperative local recurrence rate following TME is less than 10% [16]. However, lower-level TME and TME combined with abdominoperineal resection are commonly associated with a high rate of complications, as well as urinary and sexual dysfunction [17]. Over the past decade, with improvements in the diagnosis and treatment of rectal cancer, TEM has been considered an alternative to TME in the treatment of early-stage rectal cancer. Compared to TME, TEM achieves resection of rectal cancer through endoscopy, thus offering an improved field of view and leading to more precise excision of early-stage rectal cancer. In addition, TEM demonstrates a lower incidence of postoperative complications, postoperative mortality and dysfunction compared to TME [18]. However, clinical studies have reported inconsistent results in terms of the postoperative prognosis of patients receiving TEM.

There are limited numbers of trials comparing TEM and TME, and the number of RCTs is even fewer. The present study analyzed one RCT and six CCTs, focusing on the 4 aspects of local recurrence rate, distant metastasis rate, overall survival and disease-free survival. The meta-analysis conducted in the present study found insignificant differences in the rates of postoperative overall survival, disease-free survival and distant metastasis between TME and TEM. Zieren et al [19]. showed that TEM was significantly superior to TME in terms of hospital stay, complications and bleeding, and these two patient groups also did not differ in terms of survival or the rate of distant metastasis. The current study also showed that the postoperative local recurrence rate for TME was lower than that for TEM.

Numerous studies have shown that the postoperative local recurrence rate following TEM for pT1 rectal cancer is in the range of 4% to 24% [9–15, 20–22], whereas that following TME is in the range of 0% to 7% [9–15, 23]. According to recent long-term follow-up studies, the postoperative recurrence rate following TEM for the treatment of early-stage rectal cancer was higher than expected, and similar results were reported in the meta-analysis from Wu [24]. This higher-than-expected postoperative recurrence rate may be related to the following factors: 1) the trial sample size may be too small to draw authoritative results; 2) TEM is a new technique, and surgeons must strive to improve their surgical skills; 3) the location and size of rectal cancers are related to the depth of resection; and 4) after TEM, high-risk and low-risk rectal cancers differ significantly in postoperative local recurrence rates. Two articles from Borschitz [25, 26] showed that high-risk and low-risk TEM for T1 rectal cancer differed significantly in terms of the postoperative local recurrence rate and that TEM for T2 stage rectal cancer led to poor surgical results and a high local recurrence rate; in addition, secondary surgery was required to reduce the local recurrence rate to that provided by TME. Considering the TEM procedure for the management of early-stage rectal cancer is associated with a higher local tumor recurrence rate, the indications for TEM should be restricted to pT1N0M0, well (G1) or moderately (G2) differentiated rectal cancer. Results from multivariate analysis including tumor diameter, distance to anal verge and tumor differentiation will be more persuasive. Unfortunately the multivariate analysis is not applicable in this study due to the limitation that the included studies do not provide sufficient data.

Transanal excision for early rectal cancer is controversial. Recent data from well-designed observational studies have suggested high local recurrence rates and worse survival in patients treated with TAE compared to those treated with radical resection [27]. It is possible that high local recurrence rates associated with TAE may be partially related to the technical challenges of the procedure. Comparative studies of TEM and TAE for early rectal cancers demonstrated fewer resections with positive margins and lower local recurrence rates with TEM [13, 28]. A meta-analysis also concluded that TEM produced much lower local recurrence rates than transanal excision [29].

In summary, TEM demonstrated higher postoperative local recurrence rates than TME, but TEM was comparable to TME in terms of overall survival, disease-free survival and distant metastasis. The follow-up study conducted by Doornebosch [30] showed that in comparison to TME, TEM led to a more significantly improved postoperative quality of life in treated patients (particularly those who received TME combined with abdominoperineal resection). TEM is a new, minimally invasive technique with the advantages of less damage and a rapid recovery. Despite many unresolved problems, such as whether preoperative neoadjuvant chemotherapy may reduce the local recurrence rate, we believe that high-quality clinical trials with larger sample sizes may show that TEM can play an important role in the treatment of early-stage rectal cancer.

## Supporting Information

S1 PRISMA Checklist PRISMA 2009 Checklist. (DOC)

S1 Table Basic characteristics of the included studies. (DOCX)

S2 Table Result of Meta-regression. (DOCX)
