# Supplementary material for: Comparison of Transanal Endoscopic Microsurgery and Total Mesorectal Excision in the Treatment of T1 Rectal Cancer: A Meta-Analysis
Source: PLoS One. 2015 Oct 27;10(10):e0141427. doi: 10.1371/journal.pone.0141427 (PMC4624726; doi:10.1371/journal.pone.0141427)
Supplement: S1 Table — (DOCX) [file pone.0141427.s002.docx]

S1 Table Basic characteristics of the included studies

| Included studies | Treatment plan | No. of cases | Mean age (year) | Tumor diameter (cm) | Distance from the anus (cm) | Follow-up time (months) | Included indicators |
| --- | --- | --- | --- | --- | --- | --- | --- |
| Palma2009^[9]^ | TEM/TME | 34/17 | 68.4±10.7/65.6±9.7 | ≤3 | 8.9±3.2/10.9±3.5 | 86.5(48-113)/93(48-108) | ①②③④ |
| De Graaf2009^[10]^ | TEM/TME | 80/75 | 71(44-92)/67(48-83) | 3.0(0.5-13)/2.5（0.5-7.5） | 8.0(0-15)/7.0(0-15) | 42(1-127)/84(30-115) | ①②③ |
| Ptok2007^[11]^ | TEM/TME | 35/359 | 67.2(64.0-70.3)/66.3(65.3-67.4) |  |  | 42.7(39.7-45.8)/42.4(40.8-44) | ①②③ |
| Lee2003^[12]^ | TEM/TME | 52/17 | 61.1±11.2/57.7±11.8 | 2.35±0.95/3.78±1.53 | 6.7±3.2/7.5±4.0 | 31.0±17.2/34.6±19.4 | ①③④ |
| Langer2003^[13]^ | TEM/TME | 20/18 | 67.3±12.7/66.6±10.5 | 3.2±2.3/3.3±1.5 | 9.5±3/7.7±2.3 | 33.7/21.6 | ① |
| Heintz1998^[14]^ | TEM/TME | 58/45 | 66±10/64±10 | 2.5±1.5/2.9±1.6 |  | 52/42.8 | ①②③ |
| Winde1996^[15]^ | TEM/TME | 24/26 | 63.7(36-90)/60.9(47-81) |  |  | 40.9(13.3-110.6)/45.8(7.1-107.2) | ①②③ |

TEM: Transanal endoscopic microsurgery; TME: Total mesorectal excision ① Local recurrence rate ② Distant metastasis rate ③Overall survival ④ Disease-free survival
