# Supplementary material for: Comparison of Transanal Endoscopic Microsurgery and Total Mesorectal Excision in the Treatment of T1 Rectal Cancer: A Meta-Analysis
Source: PLoS One. 2015 Oct 27;10(10):e0141427. doi: 10.1371/journal.pone.0141427 (PMC4624726; doi:10.1371/journal.pone.0141427)
Supplement: S2 Table — (DOCX) [file pone.0141427.s003.docx]

S2 Table Result of Meta-regression

Meta-regression Number of obs = 7

REML estimate of between-study variance tau2 = 0

% residual variation due to heterogeneity I-squared_res = 0.00%

Proportion of between-study variance explained Adj R-squared = .%

Joint test for all covariates Model F(4,2) = 0.53

With Knapp-Hartung modification Prob > F = 0.7346

------------------------------------------------------------------------------

logor | Coef. Std. Err. t P>|t| [95% Conf. Interval]

-------------+----------------------------------------------------------------

Age | .3467099 .2742042 1.26 0.333 -.8330956 1.526515

Cases | .0003052 .0036351 0.08 0.941 -.0153353 .0159457

Follow | -.011682 .0377364 -0.31 0.786 -.1740488 .1506847

Study type | 1.257825 1.918532 0.66 0.579 -6.996953 9.512604

_cons | -22.37051 17.70814 -1.26 0.334 -98.56251 53.82149

------------------------------------------------------------------------------
